# What is health systems responsiveness? Review of existing knowledge and proposed conceptual framework

**DOI:** 10.1136/bmjgh-2017-000486

**Published:** 2017-10-31

**Authors:** Tolib Mirzoev, Sumit Kane

**Affiliations:** 1 Nuffield Centre for International Health and Development, Leeds Institute of Health Sciences, University of Leeds, Leeds, UK; 2 KIT Royal Tropical Institute, Amsterdam, The Netherlands

**Keywords:** health system, responsiveness, conceptual framework, review

## Abstract

Responsiveness is a key objective of national health systems. Responsive health systems anticipate and adapt to existing and future health needs, thus contributing to better health outcomes. Of all the health systems objectives, responsiveness is the least studied, which perhaps reflects lack of comprehensive frameworks that go beyond the normative characteristics of responsive services. This paper contributes to a growing, yet limited, knowledge on this topic. Herewith, we review the current frameworks for understanding health systems responsiveness and drawing on these, as well as key frameworks from the wider public services literature, propose a comprehensive conceptual framework for health systems responsiveness. This paper should be of interest to different stakeholders who are engaged in analysing and improving health systems responsiveness. Our review shows that existing knowledge on health systems responsiveness can be extended along the three areas. First, responsiveness entails an actual experience of people’s interaction with their health system, which confirms or disconfirms their initial expectations of the system. Second, the experience of interaction is shaped by both the people and the health systems sides of this interaction. Third, different influences shape people’s interaction with their health system, ultimately affecting their resultant experiences. Therefore, recognition of both people and health systems sides of interaction and their key determinants would enhance the conceptualisations of responsiveness. Our proposed framework builds on, and advances, the core frameworks in the health systems literature. It positions the experience of interaction between people and health system as the centrepiece and recognises the determinants of responsiveness experience both from the health systems (eg, actors, processes) and the people (eg, initial expectations) sides. While we hope to trigger further thinking on the conceptualisation of health system responsiveness, the proposed framework can guide assessments of, and interventions to strengthen, health systems responsiveness.

**Key questions**

**What is already known about this topic?**

- Responsiveness is a key objective of national health systems and is a measure of how the health system addresses legitimate expectations of individuals.
- The WHO framework is the most widely used, though there is a growing number of complementary frameworks for health systems responsiveness.
- There is, however, a shortage of alternative conceptualisation of health systems responsiveness that go beyond the normative elements of responsiveness.

**What are the new findings?**

- Health systems responsiveness entails an actual experience of people’s interaction with their health system, which confirms or disconfirms their initial expectations.
- The experience of interaction is shaped by both the people and their expectations on one side and the health systems response on the other.
- Different influences shape both the people and the system sides of interaction, ultimately affecting the resultant people’s experiences.

**Recommendations for policy**

- The proposed framework builds on and advances the core responsiveness frameworks. It positions experience of interaction as the centrepiece and recognises the determinants of responsiveness experience both from the health systems and the people’s sides.
- The framework can help advance the conceptualisation and understanding of the health systems responsiveness, inform future inquiries into systems responsiveness and guide actions to further strengthen health systems responsiveness.

## Introduction

Responsiveness, nowadays a well-recognised key objective of national health systems,^1 2^ was explicitly introduced in the World Health Report 2000.^3^ In the discussion paper which set the background for conceptualisation of health systems responsiveness by the WHO, it was defined as ‘…*when institutions and institutional relationships are designed in such a way that they are cognisant and respond appropriately to the universally legitimate expectations of individuals…*[including] *safeguarding of rights of patients to adequate and timely care’* 4 (p. 3). Better understanding health systems responsiveness is particularly important for many low-income and middle-income countries which are experiencing fast-paced economic and social development. Responsive health systems anticipate and adapt to changing needs, harness opportunities to promote access to effective interventions and improve quality of health services,^5–7^ ultimately leading to better health outcomes.^8 9^

There is a growing body of literature on health systems responsiveness, though much of it refers to responsiveness as part of other concepts. For example, responsiveness has been described as a principle of wider governance^10^ and an outcome of relationship between the people and the state^11^ or service providers.^12^ Substantial literature on accountability^13^ and acceptability and trust^14–16^ also touches on the different aspects of health systems responsiveness. Responsiveness has also been used alongside the concept of health systems resilience, for example, in the 2016 Global Health Systems Research Symposium^i^ and in the current research.^ii^ Although both responsiveness and resilience emphasise common systems characteristics such as its adaptive and transformative nature^4 17–19^ and addressing people’s needs is a key aspect of systems capacity to withstand everyday shocks and major crises,^18 20^ these are typically explored either discretely or in conjunction with broader concepts such as governance.^9 17 18 20 21^

Health systems responsiveness is a distinct, complex and not yet sufficiently explored concept.^10–15^ This perhaps explains lack of comprehensive frameworks that go beyond the normative characteristics of responsiveness of health services and also justifies the examination of responsiveness as a distinct phenomenon.

Conceptually, it includes two aspects. First is initial expectations from the people (ie, human rights bearing individuals, encompassing users and non-users of services and legal citizens and non-citizens) and the other health systems actors (most obviously, service providers and others such as managers and policy-makers) of how the individuals should be treated and within which environment.^22 23^ These expectations are likely to be shaped by social perceptions of what constitutes (ill-)health, needs, appropriate care and appropriate conduct during the care process. These expectations are shaped by characteristics of what services are available, their perceived quality and trust^16^ and the sociopolitical societal views on health as a human right.^9 24^

Second, shaped by the initial expectations, is the act of interaction itself—entailing the enactment of the multiple moments and processes of interaction between the people and the health system—shaping people’s experiences of these interactions. The most obvious point of interaction is the utilisation of health services^9^; this determines the experience of, for example, dignity, promptness or attention. Beyond the healthcare, the experiences of interaction are shaped by broader institutional arrangements, relations and interactions thereof, within the health system. These refer to, for example, the processes for people’s involvement in setting priorities or arrangements for oversight over healthcare and relations between various actors within the health system.^4 25–27^ Thus, health systems responsiveness includes a more proximal end encompassing the health services’ responsiveness (ie, how the individuals are treated) and a more distal end which is about wider system’s responsiveness (ie, the environment within which the individuals are treated).^21 22^

The most widely used framework for understanding health systems responsiveness was proposed by the WHO. It comprises seven elements against which responsiveness is measured: dignity, autonomy, confidentiality, prompt attention, quality of amenities, access to social support networks and choice of service provider.^4 25–27^ It covers different aspects of individual’s satisfaction with medical and non-medical aspects of healthcare^3 28^ and focuses on self-assessment within each element. Other frameworks represent either an extension of the WHO framework^9 21^ or focus on a specific aspect of responsiveness such as patient-provider interaction^24^ or provider accountability.^29^

In this paper, we draw on the understanding of responsiveness of public services,^21 30–34^ to add to and extend the growing, yet still limited and fragmented, knowledge on health systems responsiveness. The objective is to review, build on and extend the existing published knowledge on health systems responsiveness. More specifically, we: (1) review the current frameworks for understanding and assessing health systems responsiveness and (2) drawing on results of our review, and on key insights from the public services literature, propose a comprehensive conceptual framework for health systems responsiveness. In doing so, we also show how different concepts (satisfaction, perceived quality, rights, accountability and trust) are used, either interchangeably with or in relation to the concept of responsiveness. While we hope to trigger further thinking on the conceptualisation of health systems responsiveness, the proposed framework itself can inform future assessments, and strengthening, of health systems.

Our interest in health systems responsiveness stems from our previous analyses of policy, governance and regulation in different Asian and African contexts. We believe that this integrative piece will be of interest and relevance to different constituencies, including policy-makers and practitioners who may be interested in improving responsiveness of their health systems and academics who may be engaged in conceptualising and assessing health systems responsiveness.

The paper is structured as follows. After describing the methodology, we summarise the key frameworks for health systems responsiveness and identify the corresponding empirical work. We then reflect on key frameworks from beyond the health systems literature, to help identify the areas in which the current health systems literature can be usefully extended. Finally, we propose a comprehensive conceptual framework for understanding health systems responsiveness, building on both health systems literature and draws on key insights on service responsiveness from the public services literature.

## Methods

A comprehensive review of health systems literature was conducted. Resources from the public services literature were identified through a less structured search process.

Peer reviewed published literature was searched in April–May 2017 in three databases: OVID Medline, OVID Global Health and PubMed. The search was guided by the following keywords: health, system*, responsive*, accountab*, framework*, assess*. Following searches by individual keywords, the search results were narrowed down using three built-in filters or criteria within the databases: English-language literature, review articles and timeframe since 2000 (given the publication of responsiveness as a key health systems objective in the World Health Report 2000) and using combinations of keywords.

The search returns for individual keywords, narrowed down by review articles and the specified timeframe, resulted in about 145 000 resources and eventually the different combinations of these keywords resulted in 272 resources identified for review. The titles of each of these papers were screened for relevance to the topic and if found relevant, then further selection was based on the reading of the abstracts and then the full texts. We used the notion of a ‘framework’ flexibly and included papers which either proposed frameworks from their research (eg, by de Silva^4^ and Berlan and Shiffman^29^) or used frameworks to inform the design of their study (eg, Cleary *et al* 12). Eventually, 52 papers were selected. Although our initial intention was to focus primarily on reviews, the final selection of papers included a combination of both conceptual pieces and empirical studies which used framework(s) in assessing systems responsiveness. Some pieces have either referred to health systems responsiveness in conjunction with other concepts such as quality of care^6^ or public trust^15 16^ or explored responsiveness of selected actors to specific initiatives.^32^ While these also informed our conceptualisation of responsiveness, we do not discuss these in detail.

In addition, relevant citations from the reviewed papers were also followed up; this resulted in several additional resources (n=7) added to the review. We have also considered additional resources (n=6) which we knew about or were recommended by colleagues including an independent reviewer. Finally, a search using generic search engine (Google Scholar) was performed as an additional quality measure to ensure that no key resources were omitted.

## Results

### Current frameworks on health systems responsiveness

Eight frameworks were found in the health systems literature that focus on different aspects of health systems responsiveness (see table 1).

**Table 1:** Key frameworks for health systems responsiveness

Framework	Key points	Notes
WHO strategy, framework and tools for health systems responsiveness^4 25 28^	Health systems responsiveness is defined as experiences of health service users along the seven elements: *dignity, autonomy, confidentiality, prompt attention, quality of amenities, access to social support networks.* WHO also developed detailed survey tools and guidelines,^26^ which have been used and adapted in multiple countries.	A most widely used framework, guided world health surveys^21^ Framework and tools were used and were found applicable in different contexts^35–38 46 52^ Many studies have proposed modifications to suit the contexts of specific countries, programmes or services^34 39–42 47 57^
Three-component framework by Valentine *et al* 9	Responsiveness is viewed as a legitimate outcome of the healthcare process. It draws on the WHO framework and identifies three determinants of responsiveness: environment (the context of service provision, characteristics of welfare provision, structure of the health system and available resources) agents (users and providers) defining the need for care and setting the context process of seeking and receiving care at the individual level	No empirical studies were found, which applied this framework This framework was subsequently adapted by Robone *et al* 21 in a more detailed framework
Conceptual framework of determinants of responsiveness by Robone *et al* 21	Builds on Valentine *et al*’s framework and identifies three determinants of responsiveness: environment (resources, health systems organisation, institutional factors, eg, democratic history or corruption) characteristics of population (sociodemography, education, values) access to and use of healthcare	Framework was informed from the analysis of world health survey data
Health systems and provider responsiveness by Coulter and Jenkinson^24^	Three key components of responsiveness were identified: doctor-patient communication involvement in treatment decisions choice of provider	This framework emerged from analysis of data from a telephone survey in eight European countries
Framework for social accountability of providers by Berlan and Shiffman^29^	Two groups of determinants of social accountability of providers were identified: health system (oversight mechanisms, revenue sources and competition within the health sector—all may lead providers to be accountable to entities other than service users, eg, governments and donors) social factors (consumer power, information levels and provider beliefs surrounding accountability)	Framework emerged from synthesis of literature on health services accountability to users No empirical studies which applied this framework were found
Two-form accountability within health systems by Cleary *et al* 12	Two forms of accountability are distinguished: internal or bureaucratic (ie, within health system) external or social (to the public)	No empirical studies which applied this framework were found
SCAN Foundations Framework for advancing person-centred system of care^77^	Responsiveness is seen as an ultimate objective of healthcare system and includes five pillars: administrative reorganisation global budgeting universal assessment integrated information systems quality measurement and monitoring	Framework is aimed to inform and advance the person-centred system of care in USA
Health service responsiveness by Hashimoto *et al* 48	Specific variables of service responsiveness within vector surveillance programmes in three Latin American countries were identified as a combination of: Health systems context (distance from health centres to capitals; staff numbers, consistent monitoring, decentralised responses to vector reports) Service characteristics and outcomes (No of households reporting problems, interval between report and response)	Although framework as such was not explicitly reported, specific variables guided the data collection and analysis

As table 1 shows, the most widely used framework was proposed by the WHO in the early 2000s. The related survey toolkit was also subsequently developed.^25 26^ The framework distinguishes seven elements, along which responsiveness is self-assessed by service users.^4^

Subsequent studies have shown general agreement with the WHO elements,^35–38^ though many proposed adaptations, for example additions of effective care, attention, access to care,^39^ trust and coordination^34^ and specified it for HIV or mental health.^40–42^ Associations were also found between levels of reported responsiveness and type of health facility^37 43^ or socioeconomic characteristics of patients, such as poverty, educational level and age,^23 40 42 44–46^ echoing an argument that acceptability and trust barriers are disproportionately faced by socially disadvantaged groups. Some scholars have also suggested ranking the elements, in the contexts of either particular services such as facility-based births^46^ or health facility types such as community health centres.^47^

More recently, two inter-related frameworks—by Valentine *et al* 9 and subsequently by Robone *et al* 21—took the conceptualisation of health systems responsiveness a step further. The identification of the wider context or environment, health system and characteristics of different actors (population, service providers and others) reflects a growing recognition of wider determinants of health systems responsiveness in these two and other frameworks.^12 29 48^

An important element of health systems responsiveness, emphasised by Coulter and Jenkinson, relates to patient-provider interaction.^24^ This interaction and the resultant people’s reflections on their experiences of using services^49^—a widely recognised proxy for measuring systems responsiveness—are shaped by the characteristics of both health services (eg, availability, accessibility and quality) and people (eg, their expectations and relationships within their communities). However, in their review of provider responsiveness to social accountability initiatives, Lodenstein *et al* argued that responsiveness goes beyond the patient-provider interactions and includes ‘…*the extent to which a health provider demonstrates ‘receptivity’ to the ideas and concerns raised by citizens and to which he/she (intends to, or actually) ‘implements changes’… at the point of service’* 32 (p. 130). Such receptivity is mediated by providers’ perceptions of the legitimacy of people’s groups,^32^ provider beliefs surrounding their accountability^29^ and is likely to be influenced by the internal (health system) and external (social) forms of oversight.^12 29^

In their review, Cleary *et al* drew on Brinkerhoff and Bossert’s work on governance^13^ and on the framework proposed by Berlan and Shiffman. One argument from this body of work is that service providers are also accountable to entities other than consumers (governments and donors).^12 29^ The degree of provider accountability to other actors shapes their discretion to address people’s expectations or their receptivity to people’s concerns.^32^ All these frameworks highlight the importance of going beyond the health service-focused interpretation of responsiveness and underline the significance of interactions among providers, managers and policy-makers in shaping the system’s responsiveness.

Finally, multiple studies suggest that the conceptualisations (and the subsequent empirical assessments) of health systems responsiveness should be specific to a country context, should be cognisant of views of different actors (service users, service providers, managers), need to take account of public-private relationships, be specific to health areas such as communicable or non-communicable diseases and can be enhanced by application of a human rights approach.^32 34 38–40 43 50–52^

### Empirical assessments of health systems responsiveness

There is a growing number of alternative conceptualisations of health systems responsiveness, reflected in different frameworks summarised earlier,^12 24 29 48^ complemented by substantial theoretical and empirical literature which explored responsiveness as part of governance, accountability and trust.^5 10 11 13–16 32 53^

Three approaches to empirical assessments of health systems responsiveness can be distinguished from the literature, in relation to:

1. different components of the health system, such as public and private sectors,^43 51^ between inpatient and outpatient services^54^ or of specific cadre, such as nurses;^55^
2. specific health areas or programmes such as non-communicable diseases,^56^ HIV^57^ or mental health;^39^
3. specific population groups who are prioritised and targeted by the health system such as patients with chronic diseases^58^ or refugees.^59 60^

All empirical studies essentially attempt to measure the self-reported degree of health systems responsiveness across the different elements of responsiveness.^25 26^ Given the focus on self-assessments, the inter-related concepts of responsiveness and people’s (or specifically service users) satisfaction can be used interchangeably. However, some authors argued that these concepts have different methodological value.^22 24 61 62^ Responsiveness is considered a closer measure of actual experiences of individual’s interaction with a health system^24^ and reflecting quality of care.^6^ On the other hand, multiple influences (such as population’s sociodemographic characteristics of) were thought to play greater role in shaping people’s satisfaction, thus making it a less appropriate reflection of real service quality.^44 61 63^ In other words, both responsiveness and satisfaction are shaped by characteristics of both people and systems and differences exist in degrees to which these characteristics affect self-assessments.

Different empirical studies used, tested applicability of, and proposed adaptations to, the WHO framework.^21 26 34–42 46 47 52 57^ Consequently, different modifications were proposed to the seven elements of responsiveness, such as the addition of effective care and continuity of care to account for long-term nature of mental health services,^41 42^ treatment location and availability of information to consider specificity of HIV care^40^ or trust and coordination for chronic care.^34^ While each proposal has its merits within their respective areas, the notion of trust emerged as an important additional element of responsiveness. There is a growing body of both theoretical work^64–66^ and empirical investigations^15 67–69^ of trust within health systems, both from the patient perspective (ie, service users’ trust to health services)^34 70^ and within the health system itself (ie, trust from the perspective of service providers).^15 71^ We will return to the issue of trust later in explaining our conceptual framework.

### Responsiveness frameworks outside the health systems field

Several frameworks from literature on public services can improve understanding of health systems responsiveness.

In their conceptual piece, drawing on review of literature on public responsiveness, Liao has identified two models of responsiveness: citizen-driven model (ie, where responsiveness is primarily shaped by people’s expectations and demands) and expertise or administrator-driven model (ie, where administrators have discretionary powers to decide which expectations need responding to and which can be left unaddressed).^30^ A proposed process-driven model then attempted to bridge the citizen-driven and administrator-driven models.^30^ The administrator-driven model resembles an argument from the health systems literature that service providers can exercise discretionary powers to decide on the legitimacy of people’s expectations before addressing them.^32^ The process-driven model places the interaction between people and public services as being central to the concept of service responsiveness.

Six variants of public sector responsiveness were proposed by Bryer, from the public administration perspective.^31^ These variants, or approaches to ensuring responsiveness, are structured around three ethical perspectives (ie, competing obligations administrators typically face in their working environments):

1. *control-centred*: dictated by elected officials, constrained by systems rules, norms and procedures,
2. *discretionary*: purposive to administrator-defined goals, entrepreneurial that is, customer-oriented and
3. *deliberative*: collaborative to achieve stakeholder consensus and negotiated within multiple and conflicting demands.

The control-centred and some variants of discretionary perspectives relate to characteristics of the public system itself, whereas the customer-oriented variant and deliberative perspective focus on the interaction between customers (or service users) and the public services. In relation to health systems, these remind us of importance of interactions between people and service providers,^24^ and a conceptual demarcation between the two determinants of such interactions: health system-related (such as accountability of providers to actors other than service users^9 29^) and people-related (such as people’s socioeconomic characteristics and social accountability^21 29^).

Grove and Fisk have argued that experience of service users reflects a ‘theatre performance’ and involves ‘*the tactics and strategies employed by people to create and sustain desirable impressions before an audience’* 49 (p. 455). The authors suggest that three issues shape experiences of users of public services: actors, setting and the performance itself.^49^ All these are applicable to health systems. Actors comprise service providers, managers and policy-makers on the systems side^29^ and individual service users, their families and communities on the people’s side^32^; the setting relates to the health systems context in which health services are provided and the performance is the interaction between people and a health system (most notably at the point of health service). The performance, an important aspect of health systems responsiveness, can be understood as people’s experiences of interacting with medical and non-medical aspects of healthcare,^4 24^ which are shaped among other issues by their initial expectations.

Expectations are individuals’ predictions about what is likely to happen during an interaction. These are reference points against which people benchmark performance (of individual providers, particular services and the system at large) during their encounter with a system. In their review of literature from consumer psychology, economics and behavioural theories, Oliver and Winer differentiated between active and passive expectations.^72^ Active expectations are those which are at a high level of consciousness and are therefore instrumental in the decision to use services. In contrast, passive expectations exist as only generally true assumptions and are not processed until disconfirmed. Within health system, people may not take notice of and are indifferent to (ie, neither feel satisfied nor dissatisfied with) the health system until the interaction involves some form of confirmation of their active expectations (eg, accessibility of services) or violation of their passive expectations (eg, lack of privacy).

### Conceptual shortcomings in current approaches to health system responsiveness

Three key conceptual shortcomings can be identified in the current health systems literature. First, the notion of interaction between people and their health system, most notably at a point of service provision, is central to responsiveness. While there is substantial literature which discreetly covers the ‘health system’ and ‘people’ sides of such interaction, the interaction per se has received far less attention. This interaction and the resultant people’s service experience^49^—a proxy for measuring health systems responsiveness—are shaped by characteristics of both, the health services (eg, availability, accessibility and quality) and the people (eg, their initial expectations and relationships).

Second, while WHO’s seven elements of responsiveness are the most widely accepted measures of health system responsiveness,^4 25–27^ there is still limited recognition of its wider determinants.^21^ Existing health service encounter-focused frameworks and the resultant empirical assessments tend to under-represent or even omit the contextual determinants, such as political history and degree of democracy,^21^ characteristics of welfare provision,^9^ policy environment,^73^ available resources^21 33 73^ and characteristics of key actors (eg, users, providers, managers) which shape active and passive expectations around healthcare.^9^ These determine people’s initial expectations of the health system,^24 61 62^ thus shaping their experiences of interaction with health systems and ultimately their judgments on system’s responsiveness. These determinants also shape the health system’s ability to adequately respond to people’s expectations.

Finally, hitherto, the primary focus of key frameworks has been on responsiveness of health *services* to people’s expectations. Scholars have increasingly emphasised the key attributes and determinants of responsive health systems which relate to the health system itself, such as organisation and management of a health system^9 21^ or oversight mechanisms.^29 48^ These frameworks signal a growing recognition of importance of determinants of responsiveness which relate to the institutional arrangements, processes and relations within health systems, such as accountability between service providers, managers and policy-makers.^12^

## Discussion

We set out to review, and advance understanding of, health systems responsiveness. In this section, we discuss key knowledge gaps within the current literature on health systems responsiveness and propose a comprehensive framework to inform practice, further debate and research.

Current frameworks for health systems responsiveness, we argue, are primarily normative and focus on the ideal or desired state of people’s experience of interaction with a health system. The current empirical work has also extensively and widely engaged with, validated and nuanced these normative elements. Some frameworks articulate how the responsiveness experience is shaped by characteristics and relationships of health system (and specifically, health services); others to some extent focus on how the people-side characteristics shape their experiences.

Existing knowledge on health systems responsiveness can be usefully extended in three areas.

First, health systems responsiveness entails an actual experience of an interaction between people and their health system. This involves experience of people interacting with service providers and physical environment (eg, affecting person’s dignity, confidentiality and prompt attention) and other aspects that go beyond healthcare per se (such as quality of amenities or choice of health service provider).^4 25–27^ Therefore, health systems responsiveness is best studied as *an experience of an interaction* which people have during their encounter with a health system, be it with individuals (eg, service providers), processes (eg, service use) or organisational and institutional arrangements (eg, payment for services). Such an understanding should locate the people’s experience at the centre of health systems responsiveness.

Second, experience of people’s interaction with a health system is shaped by: (1) the people (their individual expectations and relationships within families and communities) and (2) the health system’s response (service providers and accessible and high-quality services at the ‘forefront’ and other actors, processes and institutional and organisational arrangements at the background of this interaction).^12 29–31 48^ Therefore, explicit recognition of both people and system side characteristics of interaction would enhance the conceptualisations of systems responsiveness.

Third, different influences shape both the people and the system sides of interaction. On the people’s side, examples of key influences include combination of passive and active expectations of their health systems^24 72^ and the wider historical, social, cultural and political context which shapes these expectations. On the systems side, these include attitudes of health workers^15^ and organisation and management of the health system which shape the internal accountability of service providers to actors other than service users^12 29^ and receptivity and discretion of service providers to the people’s expectations.^30 32^ Comprehensive evaluations of responsiveness need to consider all these influences.

### Proposed conceptual framework

**Figure 1:**
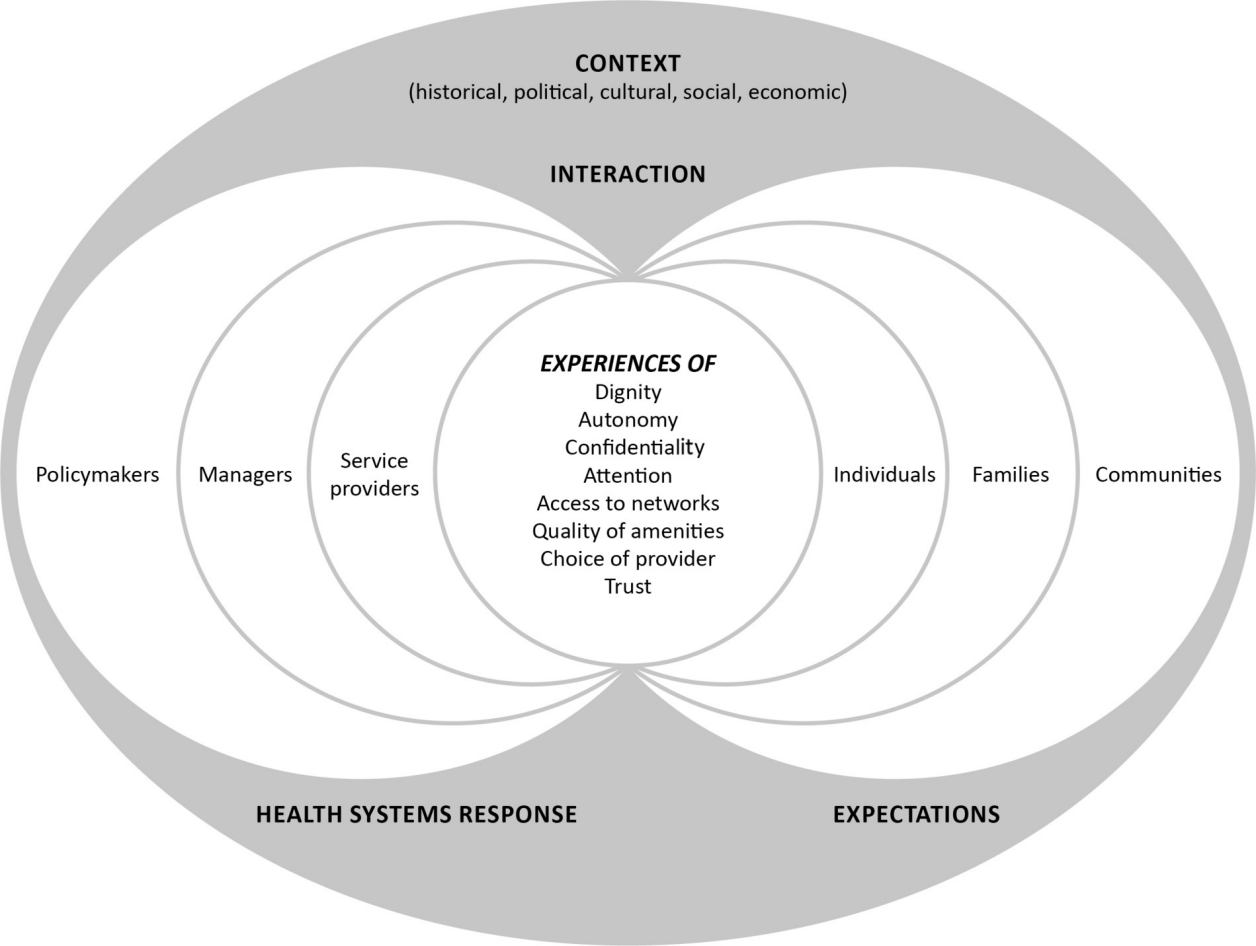
Conceptual framework for health systems responsiveness.

Synthesising insights from the current health systems and the public services literature, we now propose a comprehensive conceptual framework for health system responsiveness. Our framework (see figure 1) builds on the normative core of health systems responsiveness frameworks, including its widely accepted elements. It positions experience of people’s interaction with their health system as being central and recognises the determinants of the interaction experience both from both the health system and the people’s sides.

We locate the experience of people’s interaction with their health system at the centre of health systems responsiveness. This experience is a reflection of interaction between people and service providers^24^ at the forefront. At the background, such experience is shaped by the people’s expectations and the health systems responses to these expectations. The former recognises individual people as being part of their families and communities. However, relations between service providers, managers and policy-makers can be hierarchical, and the service providers are typically the forefront of health systems interaction with people.

As we have established earlier, people’s reflections on their experiences of interacting with their health system (and specifically health services) is a widely recognised proxy for measuring health systems responsiveness. We build on the well-accepted seven elements or measures of health systems responsiveness from the WHO framework, by adding trust—encompassing both inter-personal and institutional trust—as the eighth element of health systems responsiveness. We do so primarily responding to calls for this in the literature^34^ and in recognition of its importance in determining people’s expectations and their decisions whether to use health services.^15 34 67–69^ We also recognise that trust, and the related acceptability of health systems by people, can be interpreted as a determinant as well as an outcome of people’s interaction with their health system,^14 16^ thus potentially putting trust at the same level as responsiveness.^15^ In other words, elements of responsiveness can be interpreted as elements of people’s trust in health systems. However, we argue that trust, particularly if it is blind, can be problematic; some distrust or conditional trust, may be desirable. While high trust can spur utilisation of services, distrust, particularly if providers are aware of it, can catalyse improved systems responsiveness.^65 74^ We also acknowledge that trust can be determined by other elements of responsiveness (eg, dignity and confidentiality), though the same argument also applies to other elements of responsiveness (eg, choice of provider shapes dignity, confidentiality and attention).^14 65^

Two determinants of people’s experiences of interaction with health systems are worth noting: initial people’s expectations (shaped by their characteristics and relationships within their families and communities) and the health systems response to these expectations (eg, actors, processes, institutional and organisational arrangements, determining availability, accessibility and quality of health services). Expectations are individuals’ initial assumptions and predictions about health systems and are the benchmarks for assessing systems performance during the interaction.^49^ We echo the differentiation between the active (determinants of decision to use services) and passive (generally true assumptions which are not processed until disconfirmed) expectations.^72^ People may not take notice of and are indifferent to (ie, neither satisfied nor dissatisfied with) the health system until the interaction confirms or violates their initial expectations.

Health systems response to people’s expectations are shaped by actors, processes, institutional and organisational arrangements, including accessibility and quality of health services. Within health systems, three groups of actors can be distinguished. First, service providers, through provision of healthcare, are typically at the forefront of interaction between the people and the health system. Second, elected policy-makers and politicians define the overall direction of systems development through setting key political priorities. Third, managers and administrators (ie, civil servants) attempt to achieve the set priorities, typically through setting the standards and norms and creating processes, for example, guiding service provision. The relationships between these actors shape internal health systems accountability^12 29^ and ultimately determine the system’s receptivity to people’s expectations. That said, we recognise the importance of multiple health system’s processes, structures, resources, institutional and organisational arrangements (eg, decision-making approaches) and the resultant characteristics health services (availability, accessibility and quality), which together shape the system’s response. All these are determined, and are enacted, by key actors. Considering this and in our attempt to keep the framework simple, the health systems response in our framework is shown as only comprising the above three actor groups.

Finally, we underline the importance of the setting or the historical, political, cultural and socioeconomic context of people-system interaction. Examples of contextual influences include key political priorities,^73^ available resources and cultural norms and traditions,^21 33 73^ welfare system^9^ and specific interventions such as advocacy measures.^75^ These altogether determine the location, nature and level of services provided,^21 73^ shape the nature of organisational and professional service cultures, inform people’s expectations and frame the environment within which social relations and interactions occur between the people and their health systems.

Adaptations of this framework may be required to suit specific health areas (sectors, programmes, population groups),^39 43 51 54–57^ thus leading to further amendments of the proposed elements of responsiveness. Future assessments should go beyond the self-assessments by the people along the selected elements of systems responsiveness, as a measure of people-system interaction. Future studies should aim to better understand the people’s expectations as important determinants of their self-assessments and key influences on responsiveness from the health systems side (most notably internal accountability) as important determinants of health systems ability to respond to people’s expectations. For example, analysing system-side actors (policy-makers, managers and service providers), their roles, relations and influences should help understand receptivity and discretion of health system in addressing people’s demands.^30 32^ Analysing actors on the people side (individuals, their families and wider communities), their values, roles and relations should help understand the social environment which shapes people’s initial expectations from their health system.^72^

The proposed framework is useful across three broad areas. First, the framework can help advance the understanding of health systems responsiveness. Second, it can inform future inquiries into systems responsiveness in different contexts, focusing on both the conventional elements of responsiveness and key determinants of experiences of people-system interaction. Last, the proposed framework can guide actions to further strengthen health systems responsiveness.

### Study limitations

We acknowledge the following limitations. First, our literature search was primarily confined to the health systems literature. In an attempt to advance our understanding of responsiveness, we built on what we thought were the key frameworks from the public service literature. Therefore, a comprehensive review of the wider public services literature represents an agenda for future research. Second, our search was confined to the English-language literature. However, further bodies of knowledge exist in other languages (eg, by Shulgina in Russian^76^) which can add useful insights. Deploying multilingual teams to capture insights from multiple languages represents another area for future studies. With the above caveats, we emphasise that the overall intention of this piece was not to be exhaustive and comprehensive, but to spark further dialogue and engagement among health systems researchers and practitioners in advancing the conceptualisation of health systems responsiveness.

## Conclusion

We have reviewed existing literature on health systems responsiveness and after identifying three areas for advancing this concept, have proposed a comprehensive conceptual framework for health systems responsiveness. Our framework builds on the existing health systems literature and extends it by drawing on the published knowledge from wider public services. In our framework, we locate people’s interactions and experiences with health system as the central component. Health systems responsiveness is shaped by influences from both the people and the health system. Clarifying people’s initial expectations should help the health systems actors to adequately respond to these expectations.

## Footnotes

i Symposium website: http://healthsystemsresearch.org/hsr2016/

ii See for example: http://resyst.lshtm.ac.uk/
